# The Viral Tegument Protein pp65 Impairs Transcriptional Upregulation of IL-1β by Human Cytomegalovirus through Inhibition of NF-kB Activity

**DOI:** 10.3390/v10100567

**Published:** 2018-10-16

**Authors:** Matteo Biolatti, Valentina Dell’Oste, Sara Scutera, Francesca Gugliesi, Gloria Griffante, Marco De Andrea, Tiziana Musso, Santo Landolfo

**Affiliations:** 1 Department of Public Health and Pediatric Sciences, University of Turin, 10126 Turin, Italy; matteo.biolatti@unito.it (M.B.); valentina.delloste@unito.it (V.D.); sara.scutera@unito.it (S.S.); francesca.gugliesi@unito.it (F.G.); gloria.griffante@unito.it (G.G.); marco.deandrea@unito.it (M.D.A.); tiziana.musso@unito.it (T.M.); 2 Intrinsic Immunity Unit, CAAD–Center for Translational Research on Autoimmune and Allergic Disease, University of Piemonte Orientale, 28100 Novara, Italy

**Keywords:** human cytomegalovirus (HCMV), pp65, inflammasome, interleukin-1β (IL-1β), caspase-8

## Abstract

Interleukin-1β (IL-1β) is a key effector of the inflammasome complex in response to pathogens and danger signals. Although it is well known that assembly of the inflammasome triggers proteolytic cleavage of the biologically inactive precursor pro-IL-1β into its mature secreted form, the mechanism by which human cytomegalovirus (HCMV) regulates IL-1β production via the inflammasome is still poorly understood. Here, we show that the infection of human foreskin fibroblasts (HFFs) with a mutant HCMV lacking the tegument protein pp65 (v65Stop) results in higher expression levels of mature IL-1β compared to its wild-type counterpart, suggesting that pp65 mediates HCMV immune evasion through downmodulation of IL-1β. Furthermore, we show that enhanced IL-1β production by the v65Stop mutant is due in part to induction of DNA binding and the transcriptional activity of NF-κB. Lastly, we demonstrate that HCMV infection of HFFs triggers a non-canonical IL-1β activation pathway where caspase-8 promotes IL-1β maturation independently of caspase-1. Altogether, our findings provide novel mechanistic insights into the interplay between HCMV and the inflammasome system and raise the possibility of targeting pp65 to treat HCMV infection.

## 1. Introduction

Human cytomegalovirus (HCMV) is a ubiquitous opportunistic β-herpesvirus that is found in 50–90% of the population worldwide. Despite the occurrence of periodic reactivation and subsequent virus-shedding episodes, HCMV infection is asymptomatic in immunocompetent hosts. However, viral reactivation in immunocompromised hosts or infection of immunologically naïve fetuses in utero can lead to serious or life-threatening diseases, such as retinitis, deafness, mental retardation, malformations, and abortion [1,2].

Innate immunity is the first line of defense against HCMV, allowing the host to rapidly mount an antiviral response upon viral infection [3,4]. This is particularly prominent during the perinatal period when the immune system is still immature [3]. This innate response is mediated by type I and III interferons (IFNs) and inflammatory cytokines, such as interleukin-1β, which very rapidly create an antiviral state in the host, thereby triggering the inflammatory response [5].

An important route of innate immunity relies on the inflammasome, a multimeric protein complex that is commonly formed by sensor proteins that are known as pattern recognition receptors (PRRs), which typically sense pathogen-associated molecular patterns (PAMPs) or danger-associated molecular patterns (DAMPs), resulting in the production of proinflammatory cytokines. In this complex, the adaptor molecule that is known as apoptosis-associated speck-like proteins containing a C-terminal caspase recruitment domain (ASC or Pycard) bridges cytosolic PRRs and pro-caspase-1, with the latter being converted from an inactive zymogen into an active protease, which then catalyzes the maturation of the inflammatory cytokines interleukin-1β (IL-1β) and interleukin 18 (IL-18).

Although a plethora of stimuli, such as nucleic acids, toxins, and metabolic products [6], trigger inflammasome assembly, the mechanism of inflammasome activation and inhibition in response to viral infections still remains partly understood. Most of what we know about inflammasome modulation during cytomegalovirus (CMV) infection derives from experiments with mouse CMV (MCMV) [7,8], even though there have been few reports showing inflammasome activation in HCMV-infected monocytes or macrophages [7,8]. Among HCMV proteins, the tegument protein pp65 (pUL83) seems to play a major role in immunomodulation and immune evasion [9,10,11,12,13]. Downmodulation of caspase-1 and IL-1β activation has indeed been observed following the interaction of pp65 with the DNA sensor that is absent in melanoma 2 (AIM2) [12]. Yet, Li et al. [14] showed that pp65 can bind the pyrin domain of all nuclear pyrin and HIN domain (PYHIN) proteins (i.e., IFI16, IFIX, and MNDA), however not the AIM2-PY domain. Notably, a pp65 deletion mutant of HCMV was reportedly unable to induce inflammasome activity to levels that were comparable to that of wild-type HCMV, as judged by caspase-1 cleavage. Moreover, they found no changes in caspase-1 cleavage during HCMV infection, which led them to hypothesize that the canonical inflammasome assembly pathway does not play any role during enhanced IL-1β production upon HCMV infection [14]. Thus, despite this large body of literature, there is still much controversy surrounding the role of pp65 in the modulation of HCMV evasion mechanisms.

The aim of our study was to shed light on the molecular mechanisms of HCMV-mediated upregulation and activation of the IL-1β gene, focusing on the role of pp65. Here, we demonstrate that: (i) HCMV infection induces IL-1β expression and release, and that this effect is significantly strengthened upon infection with an HCMV pp65 mutant, which is unable to express *UL83*-encoded pp65 (v65Stop); (ii) NF-κB is a relevant transcription factor that is involved in the IL-1β promoter activation; and finally, (iii) HCMV primes IL-1β expression in a caspase-8 dependent manner.

Altogether, our results confirm and expand the key role of the tegument protein pp65 in the modulation of innate immunity.

## 2. Materials and Methods

### 2.1. Cells and Viruses

Primary human foreskin fibroblasts (HFFs, ATCC SCRC-1041™) and human embryo kidney 293 cells (HEK 293) (Microbix Biosystems Inc., Mississauga, ON, Canada) were cultured in Dulbecco’s modified Eagle’s medium (DMEM), which was supplemented with 10% fetal calf serum (FCS) (Sigma-Aldrich, Milan, Italy), as we previously described [15]. The HCMVs that were used in this study were all bacterial artificial chromosome (BAC) clones. The clones of the endotheliotropic HCMV strain TB40/E wild-type and a mutant virus that is unable to express *UL83*-encoded pp65 (v65Stop) have been described previously [16]. The viruses were propagated on HFFs and were titrated by standard plaque assay [15]. HCMV infections were all performed at a multiplicity of infection (MOI) of 1. UV-inactivated HCMV were prepared using a double pulse of UV-B light (1.2 J/cm^2^). The UV-inactivated HCMV did not replicate or produce detectable levels of immediate-early (IE) gene products.

### 2.2. Recombinant Adenoviral Vectors

The adenovirus-derived vectors (AdV) expressing pp65 were generated by means of a replacement strategy using recombineering methods as described previously [11].

### 2.3. Luciferase Assay

IL-1β promoter sequence was cloned into a luciferase pGL3-promoter vector (Promega, Madison, WI, USA). The IL-1β promoter sequences were amplified using specific sets of primers: IL-1β Fw KpnI 5′-CGGGTACCCAGCACCCAAGGTAGAGACC-3′; IL-1β Rev XhoI 5′-CGCTCGAGTGTTGGATCTTGAGGCCTAA-3′; mut-IL1-β Fw KpnI 5′- CGGGTACCTAATGTGGACATCAACTGCA-3′. The luciferase reporter constructs containing the wild-type (pIL1-β-WT) and deletion mutant (pIL1-β-NF-κB-KO) IL-1β promoter fragments and the pRL-SV40 (Promega, Madison, WI, United States) plasmid were transiently electroporated into HFFs as previously described [17]. Twenty-four hours later, they were infected with wild-type or v65Stop. Following a further 24 h post infection (hpi), firefly and Renilla luciferase activities were measured, as previously described [18], using the Dual-Luciferase reporter assay system kit (Promega, Madison, WI, USA) and a Victor X3 Multilabel Plate Reader (Perkin Elmer, Waltham, MA, USA). Firefly luciferase activity from the luciferase reporter vector was normalized to the Renilla luciferase activity from the pRL-SV40 vector. The data report the ratio of relative light units (RLU) that were measured for firefly luciferase activity to the RLU that were measured for Renilla luciferase activity.

### 2.4. RNA Isolation and Semiquantitative RT-qPCR

Total RNA was extracted using the NucleoSpin RNA kit (Macherey-Nagel, Düren, Germany) and 1 μg was retrotranscribed using the Revert-Aid H-Minus FirstStrand cDNA Synthesis Kit (Thermo Fisher Scientific, Waltham, MA, USA), according to the manufacturer’s protocol. The comparison of mRNA expression between the samples (i.e., infected versus untreated) was performed by SYBR green-based RT-qPCR using Mx3000P apparatus (Stratagene, San Diego, CA, USA) using the following primers: IL-1β Fw TCCCCAGCCCTTTTGTTGA, IL-1β Rw TTAGAACCAAATGTGGCCGTG; the housekeeping gene Glyceraldehyde-3-phosphate dehydrogenase (GAPDH) Fw AGTGGGTGTCGCTGTTGAAGT, GAPDH Rw AACGTGTCAGTGGTGGACCTG.

### 2.5. Immunofluorescence Microscopy

Indirect immunofluorescence analysis was performed as previously described [19]. The following primary antibodies were used: rabbit polyclonal anti-IEA (Santo Landolfo, University of Turin, Italy), and mouse monoclonal anti-NF-κB p65 (Santa Cruz Biotechnology, Dallas, TX, USA). Signals were detected using goat anti-rabbit or goat anti-mouse conjugated secondary antibodies (Thermo Fisher Scientific, Waltham, MA, USA). Nuclei were counterstained with 4′,6-diamidino-2-phenylindole (DAPI). The samples were observed using a fluorescence microscope (Olympus IX70, Olympus Italia, Segrate, Italy) that was equipped with cellSens Standard-Microscopy Imaging Software. ImageJ software was used for image processing.

### 2.6. Enzyme-Linked Immunosorbent Assay (ELISA)

Cell-free supernatants were harvested and IL-1β production was measured by DuoSet ELISA assay according to the manufacturer’s instructions (R&D Systems, Minneapolis, MN, USA). All absorbance readings were measured at 450 nm using an ELISA Plate Reader (DAS, Palombara Sabina, Italy).

### 2.7. Non-Radioactive Universal EZ-TFA Transcription Factor Assay

The DNA binding activity of p65/RelA was measured using a Universal EZ-TFA transcription factor assay colorimetric kit (Upstate Biotechnology Inc., Lake Placid, NY, USA) according to the manufacturer’s protocol. In brief, a double-stranded biotinylated oligonucleotide containing the consensus sequence for p65 was used as a capture probe; it was mixed with nuclear extracts and was added directly into the streptavidin-coated plate. An unlabeled oligonucleotide containing the identical consensus sequence as the capture probe was used as a competitor. Any inactive, unbound material was washed away and the bound p65 was detected with a specific primary antibody. A horseradish peroxidase (HRP)-conjugated secondary antibody was then used for detection, and p65 specific binding was quantified at 450 nm using a microplate reader. The p65 probe sequences were: sense (biotin): 5′-ATGACATAGGAAAACTGAAAGGGAGAAGTGAAAGTGGGAAATTCCTCTG-3′; antisense: 5′-CAGAGGAATTTCCCACTTTCACTTCTCCCTTTCAGTTTTCCTATGTCAT-3′. Unlabeled oligonucleotide was added as the competitor DNA.

### 2.8. In Vitro Analysis of Caspase-1 and Caspse-8 Activity

Caspase-1 and caspase-8 protease activity was measured by evaluating the extent of cleavage of a fluorometric peptide substrate using SensoLyte AFC Caspase Sampler Kit Fluorimetric (Anaspec, CA, USA). The experiments were performed according to the manufacturer’s instructions. After an hour of incubation at 25 °C, fluorescence was measured at an excitation wavelength of 405 nm and an emission wave length of 500 nm using the Victor X3 Multilabel Plate Reader (Perkin Elmer, Waltham, MA, USA). Protease activity was expressed as the fold induction of HCMV-infected vs. mock-infected cells.

### 2.9. Inhibition of Caspase-1 and Caspase-8 Expression

HFFs were transiently transfected with a MicroPorator (Digital Bio Pharm, London, Great Britain) according to the manufacturer’s instructions (1200 V, 30 ms pulse width, one impulse) with a pool of small interfering RNAs (Qiagen, Hilden, Germany) targeting caspase-1 (siCASP1, FlexiTube siRNAs cat. No.: SI0266244304132170, SI0266193204263189, SI0265459604287626, SI0494828604357696), caspase-8 (siCASP8, FlexiTube siRNAs cat. No.: SI0266245704359754, SI0266194604210101, SI0029959304164797, SI0494831404131687), or control siRNA (siCTRL, 1027292) as negative control.

### 2.10. Statistical Analysis

All statistical tests were performed using GraphPad Prism version 5.00 for Windows (GraphPad Software, San Diego, CA, USA). The data were presented as means ± standard deviations (SD). Statistical significance was determined by using two-tailed Student’s *t*-tests; one-way or two-way analysis of variance (ANOVA) with Bonferroni’s post-tests, as appropriate. Differences were considered statistically significant for *p* < 0.05 (*p* < 0.05 *; *p* < 0.01 **; *p* < 0.001 ***).

## 3. Results and Discussion

### 3.1. IL-1β Induction upon HCMV Infection is Inhibited by the HCMV Tegument Protein pp65 in an NF-κB Dependent Manner

**Figure 1:**
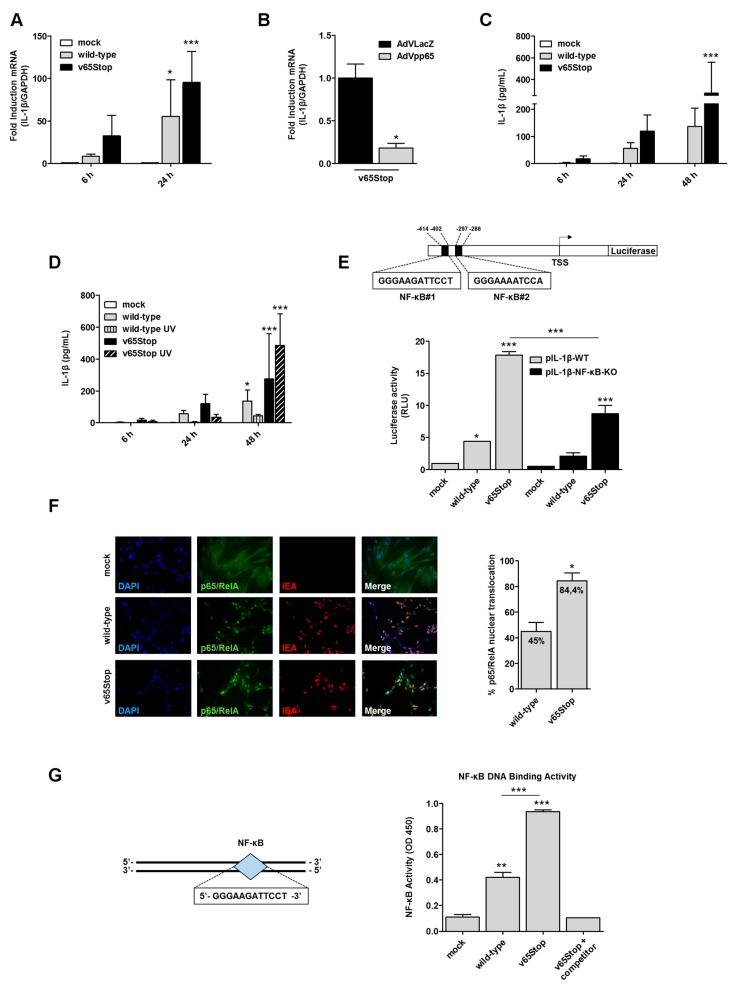
HCMV (human cytomegalovirus) pp65 inhibits IL-1β (interleukin-1β) response following HCMV infection of HFFs (human foreskin fibroblasts) through NF-κB. (**A**) HFFs were infected at an MOI of 1 with wild-type or v65Stop HCMV and were analyzed by RT-qPCR. IL-1β mRNA expression levels following HCMV vs. mock infection were normalized to glyceraldehyde-3-phosphate dehydrogenase (GAPDH) and are shown as mean fold changes ± SD (*, *p* < 0.05; ***, *p* < 0.001; two-way ANOVA followed by Bonferroni’s post-tests). (**B**) HFFs were transduced with AdVLacZ (black bar) or AdVpp65 (grey bar) at an MOI of 50. Subsequently, cells were infected with v65Stop HCMV (MOI of 1). At 15 hpi, IL-1β mRNA expression was normalized to that of GAPDH and is shown as a mean ± SD fold change (*, *p* < 0.05; unpaired *t*-test). (**C**) HFFs were infected with wild-type or v65Stop HCMV at an MOI of 1. Supernatants were collected at 6, 24, and 48 hpi and were assessed by ELISA for IL-1β production. Results are shown as a mean ± SD fold change (***, *p* < 0.001; two-way ANOVA followed by Bonferroni’s post-tests). (**D**) HFFs were infected with wild-type, wild-type UV, v65Stop, or v65Stop UV at an MOI of 1. Supernatants were collected at 6, 24, and 48 hpi and were assessed by ELISA for IL-1β production. Results are shown as the mean ± SD fold change (*, *p* < 0.05; ***, *p* < 0.001; two-way ANOVA followed by Bonferroni’s post-tests). (**E**) Schematic representation of the IL-1β luciferase promoter plasmid with the sequences containing the two putative NF-κB binding sites indicated as NF-κB#1 and NF-κB#2 (left panel). HFFs were transiently electroporated with luciferase plasmids encoding the wild-type (pIL-1β-WT) or deletion mutant (pIL-1β-NF-κB-KO) IL-1β promoter fragments, and pRL-SV40. Twenty-four hours later, the cells were mock-infected or infected with wild-type or v65Stop HCMV at an MOI of 1. At 24 hpi, firefly and Renilla luciferase activities were measured. The luciferase activity in whole-cell lysates was normalized to Renilla luciferase activity and is expressed as relative light units (RLU) (right panel). Results are shown as the mean ± SD fold change (*, *p* < 0.05; ***, *p* < 0.001; one-way ANOVA followed by Bonferroni’s post-tests). (**F**) HFFs mock-infected or infected with wild-type or v65Stop HCMV at an MOI of 1 were fixed at 24 hpi and were subjected to immunofluorescence analysis. HCMV-IEA (red) and p65/RelA (green) were visualized using primary antibodies, followed by secondary antibody staining, in the presence of 10% HCMV-negative human serum. Nuclei were counterstained with DAPI (4′,6-diamidino-2-phenylindole) (blue). The graph (right panel) shows the levels of nuclear NF-κB in infected cells. The data represent the mean fold changes (*, *p* < 0.05; unpaired *t*-test). (**G**) Schematic representation of the probe containing the NF-κB#1 binding site (left panel). HFFs were left untreated (mock) or infected with wild-type or v65Stop (MOI of 1). At 24 hpi, the cells were lysed and the nuclear fraction was analyzed for NF-κB binding activity using the Universal EZ-TFA transcription factor assay colorimetric kit (right panel). The data show the means ± SD (**, *p* < 0.01; ***, *p* < 0.001; one-way ANOVA followed by Bonferroni’s post-test).

HCMV infection induced the production of IL-1β in different cell types [20,21,22]. Moreover, we and others have identified the HCMV tegument protein pp65 [9,10,11,12] as one of the key mediators of HCMV evasion from the innate immune response. Thus, we sought to determine whether the immunosuppressive function of pp65 could be mediated by the inflammasome system. To this end, HFFs were first mock-infected or infected with wild-type HCMV or v65Stop HCMV, a mutant that is unable to express *UL83*-encoded pp65 [11,16], at an MOI of 1, and total RNA was analyzed by RT-qPCR at 6 and 24 hpi. HCMV-IEA mRNA expression was employed as a positive control for viral infection (data not shown). As shown in Figure 1A, IL-1β mRNA levels that were observed at 24 hpi in v65Stop-infected HFFs were approximately 1.7-fold higher than those that were observed in cells that were infected with wild-type HCMV, indicating that pp65 may negatively regulate *IL-1β* gene expression.

To test whether ectopic expression of pp65 would downregulate IL-1β gene expression, we either infected HFFs constitutively expressing pp65 protein (AdVpp65) or not (AdVLacZ) with v65Stop HCMV for 15 h. Consistent with our previous results, RT-qPCR analysis revealed that IL-1β mRNA expression levels were reduced by approximately 80% in AdVpp65-infected cells compared to the control (Figure 1B). Thus, these findings suggest a model whereby pp65 plays an essential role in HCMV escape from the host immune response through the downmodulation of IL-1β gene expression.

Next, we asked whether enhanced levels of IL-1β mRNA in v65Stop-infected HFFs would correlate with increased production of biologically active IL-1β protein. For this purpose, supernatants from HFFs that were infected with wild-type or v65Stop viruses were harvested at 6, 24, and 48 hpi and were assessed by ELISA for IL-1β production. As shown in Figure 1C, and consistent with the results obtained with RT-qPCR, the levels of IL-1β that were secreted at 24 and 48 hpi are considerably higher in v65Stop HCMV-infected cells than those that were observed in wild-type HCMV-infected ones, indicating that in the presence of HCMV pp65, the signaling pathway leading to IL1-β production is impaired (Figure 1C). After viral envelope fusion, the virion-associated pp65 is released into the cytoplasm and subsequently translocates to the cell nucleus. To determine whether pp65 protein delivery is sufficient for downmodulation of IL-1β production or whether viral gene expression is required, we employed the UV-inactivated viruses wild-type and v65Stop (named wild-type UV and v65Stop UV). In detail, supernatants from HFFs that were infected with wild-type, wild-type UV, v65Stop, and v65Stop UV viruses were harvested at different times post infection (6–48 hpi) and were assessed by ELISA for IL-1β production. As shown in Figure 1D, the levels of secreted IL-1β are significantly lower in the wild-type and wild-type UV -infected cells than those that were observed in v65Stop and v65Stop UV, and this effect is more appreciable at 48 hpi. Overall, these results support our hypothesis that virion-associated pp65 released in the cytoplasm in the early stage of infection contributes significantly to the modulation of IL-1β production (Figure 1D).

Since HCMV infection results in NF-κB dysregulation [9,23,24,25], and the IL-1β promoter contains two putative binding sites for the NF-κB transcription factors, located at positions −412 to −402 (5′-GGGAAGATTCCT-3′) and -297 to -286 (5′-GGGAAAATCCA-3′) (Figure 1E) [26], we sought to determine whether downmodulation of IL-1β gene expression occurred at the transcriptional level due to inhibition of NF-κB activity. For this purpose, the luciferase reporter constructs containing the wild-type (pIL1-β-WT) and deletion mutant (pIL1-β-NF-κB-KO) IL-1β promoter fragments were transiently transfected into HFFs. Twenty-four hours later, the cells were left uninfected or infected with wild-type or v65Stop HCMV. Luciferase activity was then assessed following an additional 24 h of incubation. As shown in Figure 1E, v65Stop HCMV-infected HFFs display a 4-fold induction of pIL-1β-WT luciferase activity compared to that of the cells that were infected with wild-type HCMV. By contrast, the induction of pIL-1β-NF-κB-KO luciferase activity was decreased by approximately 50% in the cells that were infected with either v65Stop or wild-type HCMV. Thus, deletion of NF-κB binding sites downregulates IL-1β promoter activity following HCMV infection, suggesting that the downregulation of IL-1β gene expression by v65Stop HCMV occurs at the transcriptional level and that it is likely mediated by inhibition of NF-κB activity. However, the residual activity of IL-1β promoter observed in pIL-1β-NF-κB-KO suggests that other transcription factors may be involved in the regulation of IL-1β promoter activity.

NF-κB is a heterodimer consisting of a 50-kDa subunit (p50) and a 65-kDa subunit (p65/RelA). Under normal physiological conditions, NF-κB is sequestered in the cytosol by a family of IκB inhibitors in an inactive state [9,23,27]. To ascertain whether HCMV infection promotes RelA translocation to the nucleus and whether this effect is opposed by pp65, HFFs were infected with wild-type or v65Stop for 24 h and were analyzed by indirect immunofluorescence using anti-RelA antibodies. As shown in Figure 1F, 45% of wild-type infected cells display RelA nuclear translocation. By contrast, and consistent with the results reported by Browne et al. [9], nuclear translocation of RelA was more pronounced in cells that were infected with v65Stop (84.4% of infected cells), indicating a stronger activation of NF-κB in the absence of HCMV pp65.

Upon its translocation to the nucleus, RelA binds to specific response elements in the promoter regions of responsive genes. Having observed that pp65 impairs the nuclear translocation of RelA, we next sought to determine whether pp65 could inhibit DNA binding of RelA to its consensus site, thereby preventing its transcriptional activity. To this end, we took advantage of the Universal EZ-TFA transcription factor colorimetric assay, which combines the DNA-binding principle of the electrophoretic mobility shift assay with the 96-well format of an ELISA assay [28,29]. In this context, nuclear extracts from v65Stop HCMV-infected cells exhibited an approximately 50% increase in RelA DNA-binding activity compared to nuclear extracts from HFFs that were infected with wild-type HCMV at 24 hpi (OD 450 of p65—DNA binding, wild-type vs. v65Stop HCMV infected cells: 0.4 vs. 0.9) (Figure 1G). Altogether, these results demonstrate that pp65 inhibits IL-1β gene expression at the level of transcription by reducing binding of RelA to at least one of the two κB sites within the IL-1β promoter.

### 3.2. HCMV Primes IL-1β Activation in a Caspase-8 Dependent Manner

The maturation step of bioactive IL-1β has long been thought to be catalyzed solely by the inflammatory cysteine protease caspase-1, also known as ICE (IL-1β-converting enzyme) [30]. However, recent evidence indicates alternative pathways of IL-1β release independent of inflammasome-induced caspase-1 activation. In this regard, caspase-8 has recently been identified as an alternative protease that can process IL-1β independently of the inflammasome leading to the secretion of the active form [30,31,32]. Furthermore, studies in HEK293T cells overexpressing both caspase-8 and pro-IL-1β have shown that caspase-8 can directly cleave pro-IL-1β in response to stimulation by TLR3 or TLR4. Furthermore, stimulation of dectin-1, a macrophage-specific C-type lectin receptor, has been shown to lead to caspase-8 activation and caspase-1-independent maturation of pro-IL-1β [32].

**Figure 2:**
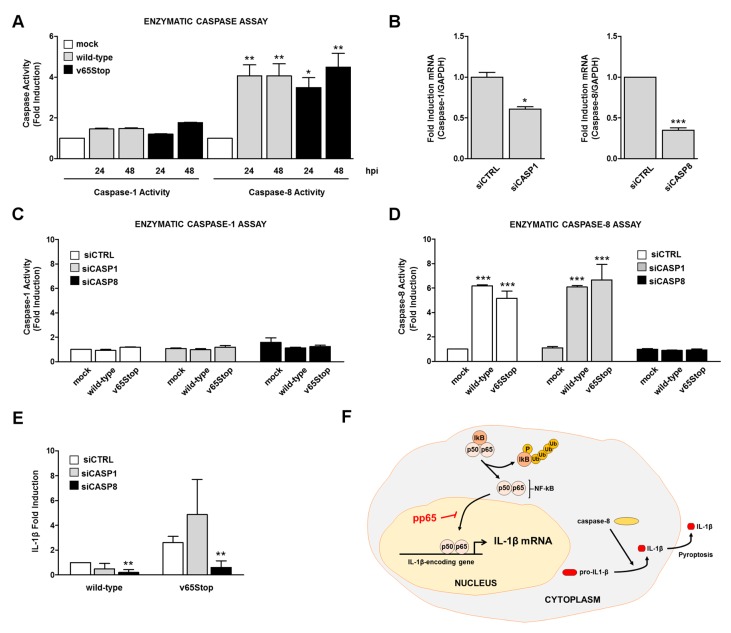
HCMV primes IL-1β activation in a caspase-8 dependent manner. (**A**) HFFs were infected with wild-type or v65Stop HCMV at an MOI of 1 for 24 and 48 h and were processed by fluorimetric assay for caspase-1 and caspase-8 activation. Fold changes were calculated after normalization of HCMV vs. mock-infected cells. Data are shown as mean ± SD (*, *p* < 0.05; **, *p* < 0.01; one-way ANOVA followed by Bonferroni’s post-tests). (**B**–**D**) HFFs were electroporated with pools of siRNA targeting caspase-1 (siCASP1), caspase-8 (siCASP8), or scrambled control siRNA (siCTRL). (**B**) The efficiency of caspase-1 and caspase-8 depletion was assayed by RT-qPCR (the data are shown as mean fold changes plus SD; *, *p* < 0.05; ***, *p* < 0.001; by unpaired *t*-test). (**C**,**D**) siCTRL, siCASP1, and siCASP8 HFFs were infected with wild-type or v65Stop HCMV at an MOI of 1 and were examined at 24 hpi by fluorimetric assay for caspase-1 (**C**) and caspase-8 (**D**) activation. Results are shown as the mean ± SD fold change (***, *p* < 0.001 two-way ANOVA followed by Bonferroni’s post-tests). (**E**) Cells were infected as described in (**C**,**D**). Supernatants were collected at 24 hpi and were analyzed by IL-1β ELISA. Results are shown as the mean ± SD fold change over wild-type siCTRL set as 1.0. A statistically significant difference compared to siCTRL is indicated by asterisks (**, *p* < 0.05; unpaired *t*-test). (**F**) Model depicting the proposed functional role of pp65 modulation of IL-1β activity during HCMV infection.

Based on these findings, we compared the activity of caspase-1 and caspase-8 at different time points after infection with wild-type or v65Stop HCMV-infected HFFs. In agreement with Li et al. [14], we failed to detect changes in caspase-1 activity under any conditions, suggesting that caspase-1 activity does not play a role during inflammasome assembly following HCMV infection (Figure 2A). By contrast, we observed a significant activation of caspase-8 activity in HFFs that were infected with either HCMV strains compared to the mock (Figure 2A). Interestingly, there was no significant difference in the ability to activate caspase-8 between the two virus strains, suggesting that HCMV pp65 decreases IL-1β at the transcriptional level, however that it does not interfere with caspase activation.

It is relevant to know that HCMV *UL36* encodes for a cell death suppressor, named pUL36 or vICA, which binds to the pro-domain of caspase-8 and prevents its activation, thereby blocking the Fas-mediated apoptosis-signaling pathway [33]. In addition, Skaletskaya and colleagues [33] showed that a strain-specific single point-mutation observed in AD169*var*ATCC (Cys ^131^ → Arg ^131^) affects the binding of vICA to pro-caspase-8 and consequently abrogates its antiapoptotic activity.

In order to assess whether the two HCMV strains that were used in our experiments carried an intact *UL36* gene, *UL36* regions were sequenced and were compared with the published sequences from TB40/E, AD169*var*ATCC, and the Towne strain, which was shown to induce caspase-8 activity in HFFs at later time points after infection [34]. An alignment of the *UL36* sequences from the different HCMV strains confirmed that our HCMV strains do not carry the mutation found to render pUL36 inactive, suggesting that at earlier time points of the infection, pUL36 could still be able to inhibit caspase-8 activity, however that a residual amount of pro-caspase-8 gets activated and cleaves IL-1β (data not shown).

To strengthen the specificity of these results, we performed ablation experiments using a mixture of specific siRNAs targeting caspase-1 (siCASP1), caspase-8 (siCASP8), or scrambled control siRNA (siCTRL). The efficacy of silencing was assessed by RT-qPCR (Figure 2B) and by caspase activity (Figure 2C,D). As shown in Figure 2C, no caspase-1 activity was detected under any conditions. By contrast, siCTRL- and siCASP1-treated cells showed increased caspase-8 activity in response to HCMV infection (Figure 2D).

Next, to assess more directly the involvement of caspase-1 and/or caspase-8 in IL-1β maturation, we performed an ELISA specific for IL-1β using supernatants that were obtained from HFFs that were transiently depleted of caspase-1 or caspase-8, respectively, and were then infected with wild-type or v65Stop HCMV for 24 h. A significant decrease in IL-1β production was observed in cells that were depleted for caspase-8, however not caspase-1, which were then infected with wild-type or v65Stop HCMV (Figure 2E), indicating that caspase-8 is necessary for maturation of IL-1β during HCMV infection. In agreement with the enzymatic activity assay, a residual induction of IL-1β release was still present in HCMV-infected caspase-8-deficient HFFs compared to siCTRL- or siCASP1-treated cells, implying that other factors may contribute to IL-1β production. Altogether, these results indicate that caspase-8 is essential to mount an abundant IL-1β maturation in response to HCMV infection.

## 4. Conclusions

Our findings add novel functional and mechanistic insights into the role that is played by the HCMV tegument protein pp65 in HCMV immune evasion. In this regard, we show that the production of mature IL-1β is significantly increased upon infection with an HCMV mutant that is unable to express pp65. Furthermore, the observation that the infection of HFFs with the v65Stop HCMV mutant leads to the induction of both NF-κB DNA binding and transcriptional activities at promoter regions of IL-1β, thereby enhancing the production of IL-1β, strongly argues in favor of an inhibitory role that is played by HCMV pp65 during IL-1β production. Equally important, we show for the first time that a non-canonical pathway has evolved in response to HCMV infection, leading to inflammasome-independent maturation of IL-1β via caspase-8 activation (Figure 2F).

Overall, our studies further confirm and expand the prominent role of the tegument protein pp65 in the modulation of the innate immune response, a function that could be exploited therapeutically.
